# Explainable machine learning model predicts response to adjuvant therapy after radical cystectomy in bladder cancer

**DOI:** 10.3389/fonc.2025.1664965

**Published:** 2025-10-31

**Authors:** Jian Hou, Yi Ding, Runlin Feng, Yumin Wang, Yanping Tao, Junxiong Li, Jingbo Qin, Pinyao Liang, Peng Gu, Xiaodong Liu

**Affiliations:** ^1^ Department of Urology, The First Affiliated Hospital of Kunming Medical University, Kunming, Yunnan, China; ^2^ Department of Urology, The Third Affiliated Hospital of Wenzhou Medical University, Wenzhou, Zhejiang, China; ^3^ Department of Pathology, The Second Affiliated Hospital of Kunming Medical University, Kunming, Yunnan, China; ^4^ Department of Emergency Medicine, Kunming Third People’s Hospital, Kunming, Yunnan, China

**Keywords:** bladder cancer, adjuvant therapy, machine learning, shap, predictive model, radical cystectomy, molecular markers

## Abstract

**Purpose:**

Radical cystectomy (RC) is the standard treatment for muscle-invasive and select high-risk non–muscle-invasive bladder cancer. Despite definitive surgery, recurrence and progression remain major clinical concerns. Adjuvant chemotherapy and immunotherapy may improve outcomes, but therapeutic response varies due to tumor heterogeneity. Robust predictive models are needed to guide individualized treatment strategies.

**Methods:**

This study retrospectively analyzed bladder cancer patients undergoing RC. Data included tumor morphology (e.g., vascular and perineural invasion), demographic variables (e.g., age, sex), and molecular markers (e.g., PD-L1, HER2, GATA3). LASSO regression identified key features, followed by model development using nine machine learning algorithms, including XGBoost and LightGBM. Model performance was assessed via area under the ROC curve (AUC), and Shapley Additive Explanations (SHAP) were used for model interpretability.

**Results:**

The random forest model achieved the highest predictive performance (AUC = 0.92 in training; 0.74 in testing). SHAP analysis identified vascular invasion, perineural invasion, and PD-L1/HER2 expression as major contributors. Decision curve analysis showed favorable net benefit within a moderate-risk threshold.

**Conclusions:**

A machine learning model integrating pathological, demographic, and molecular features demonstrates promising potential to predict response to adjuvant therapy post-RC in bladder cancer. Decreased performance in the external test cohort highlights the need for further validation. Prospective studies incorporating multi-center and longitudinal data are warranted to enhance model generalizability and clinical applicability.

## 1 Introduction

Bladder cancer ranks among the most common malignancies globally, with approximately 573,000 new cases and 213,000 deaths reported each year (1). Radical cystectomy (RC) remains the standard treatment for muscle-invasive bladder cancer (MIBC) and high-risk non-muscle-invasive bladder cancer (NMIBC). However, postoperative recurrence and disease progression continue to be major clinical challenges. To improve patient outcomes, adjuvant therapies—including chemotherapy, immunotherapy, and targeted therapy—have been increasingly utilized in clinical practice (2). Adjuvant chemotherapy (AC) is recommended for patients with high-risk MIBC and NMIBC following RC, but its efficacy is not always predictable, and the selection of patients for adjuvant therapy remains a clinical challenge.Neoadjuvant chemotherapy (NAC) is currently recommended prior to RC in patients with MIBC. Although NAC offers a 5–10% improvement in overall survival, not all patients derive benefit. Identifying non-responders is therefore essential to avoid unnecessary toxicity and delays in definitive surgery (3).

In this study, adjuvant chemotherapy is specifically recommended for patients with pathological stages of T3 or higher, positive surgical margins, or evidence of vascular/perineural invasion. These factors significantly increase the risk of recurrence and progression, justifying the need for adjuvant therapy after radical cystectomy (RC). Traditional adjuvant chemotherapy selection relies on clinical experience and factors like tumor stage and grade. However, the unpredictable nature of treatment responses highlights the need to identify patients who will benefit from adjuvant chemotherapy, while avoiding unnecessary toxicity. Machine learning techniques, integrating complex, multi-dimensional data, provide a promising solution to personalize treatment selection and improve patient outcomes.

In recent years, machine learning (ML) has shown great potential in predicting the response to neoadjuvant and adjuvant treatment in bladder cancer by integrating clinical, pathological, and molecular data. Deep learning models, for instance, have been used to predict survival outcomes in MIBC patients treated with NAC, demonstrating superior predictive performance over traditional statistical models (4). Similarly, ML-based prognostic models have been applied to assess response to immunotherapy (5). Tumor mutational burden (TMB)-based classifiers, developed using support vector machine recursive feature elimination (SVM-RFE) and LASSO logistic regression, have also been employed to predict the efficacy of PD-L1 inhibitors in patients with locally advanced or metastatic urothelial carcinoma (6). Despite these advances, the substantial heterogeneity of bladder cancer leads to variable treatment responses, and no robust predictive model currently exists to evaluate response to adjuvant therapy following RC. Thus, this study focuses on the development of predictive models for adjuvant chemotherapy, aiming to address the gap in personalized treatment strategies for post-surgical bladder cancer patients.

Over the past decade, several prognostic models for bladder cancer have been proposed, primarily based on clinicopathological features such as tumor stage, histological grade, and lymph node involvement (7–9). However, these traditional models often rely on single-dimensional data and linear statistical methods, limiting their predictive accuracy. Recent advances in artificial intelligence have enabled the integration of multimodal data—including tumor morphology, molecular biomarkers, and demographic factors—resulting in improved predictive performance (10). Among ML algorithms, extreme gradient boosting (XGBoost), random forest (RF), and light gradient boosting machine (LightGBM) have shown strong capabilities in handling high-dimensional data, identifying complex variable interactions, and enhancing predictive performance, and have been widely applied across various cancer types.

The role of molecular biomarkers in predicting adjuvant treatment response in bladder cancer is gaining increasing attention. Immune checkpoint molecules such as PD-L1, as well as oncogenic markers including HER2 and androgen receptor (AR), have been shown to be of significant prognostic value in predicting therapeutic responses (11–13). Moreover, components of the tumor immune microenvironment—such as macrophage infiltration and immune checkpoint expression—have been implicated in treatment resistance (14, 15), suggesting that predictive models incorporating histopathological, molecular, and demographic characteristics may offer more accurate risk stratification.

**Figure 1:**
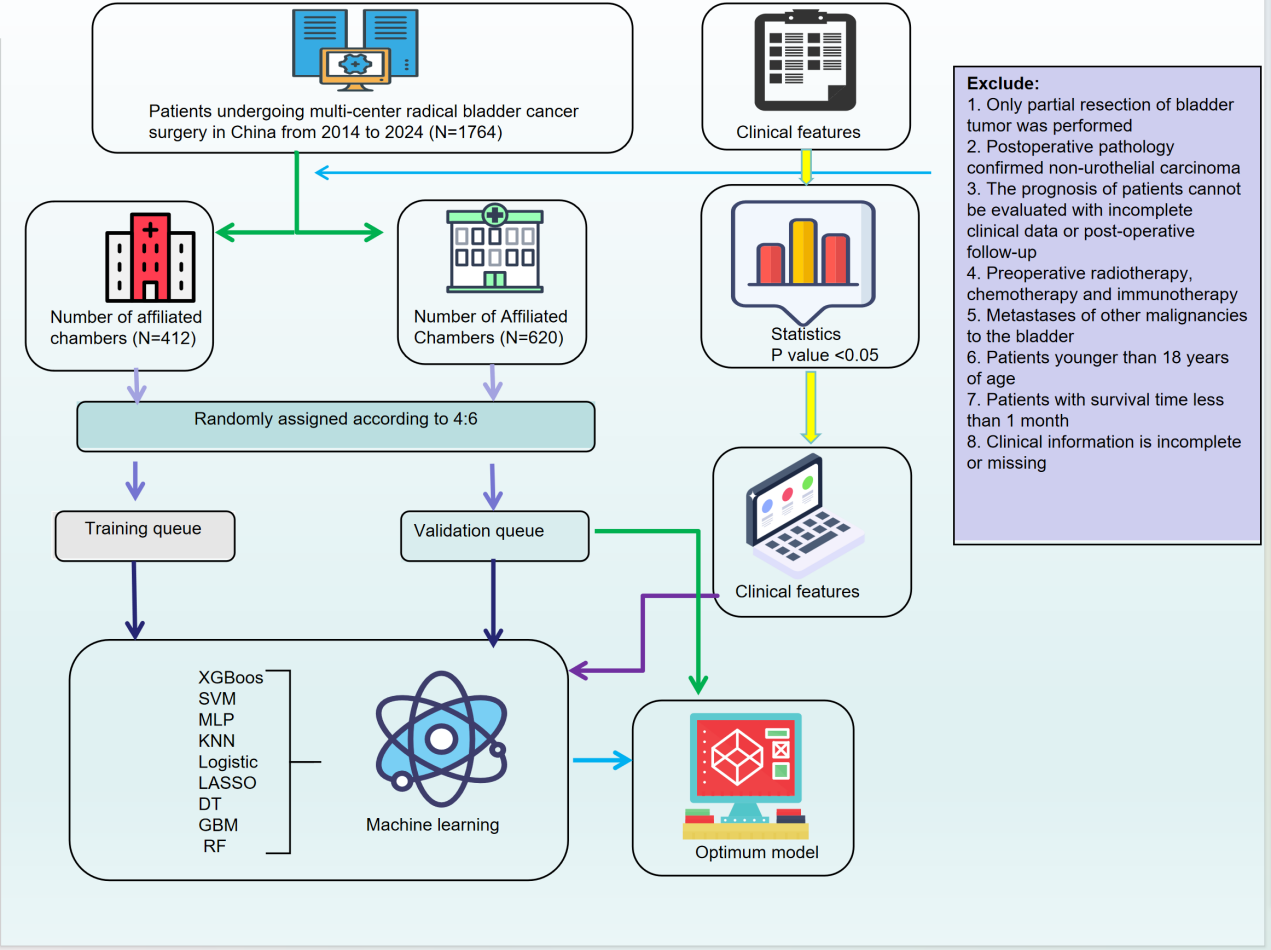
Workflow of model development and validation.

This study aims to develop and validate a machine learning-based predictive model integrating tumor morphological features, demographic variables, and molecular marker expression to assess the response of bladder cancer patients to adjuvant therapy following radical cystectomy. By employing advanced feature selection techniques and multiple ML algorithms, we seek to establish an optimized predictive framework to support clinical decision-making and promote individualized therapeutic strategies.Furthermore, we focus on improving the accuracy of adjuvant therapy selection, which has traditionally relied on clinical experience and single-dimensional factors. Incorporating machine learning can better address this gap and personalize treatment choices, minimizing unnecessary toxicity and improving patient outcomes. In addition, SHapley Additive exPlanations (SHAP) analysis is applied to improve model interpretability and to explore the contribution of key features to treatment outcomes. The results of this study may have significant clinical implications for optimizing adjuvant therapy in bladder cancer, improving patient survival, and minimizing treatment-related toxicity (**Figure 1**).

## 2 Materials and methods

### 2.1 Data collection and processing

We retrospectively collected clinical data from 1,764 bladder cancer patients who underwent radical cystectomy (RC) between 2014 and 2024 at the First and Second Affiliated Hospitals of Kunming Medical University. The dataset included demographic characteristics (age, sex, ethnicity, smoking status, alcohol consumption), medical history, tumor morphology, and pathological features (e.g., Uroplakin-III, PD-L1, HER2, perineural invasion). In addition to these features, pathological staging, tumor grade, and surgical margin status were also integral to the dataset. Patients were categorized based on pathological stage, specifically focusing on stages T3 and higher, with particular attention to surgical margin involvement and evidence of vascular or perineural invasion. These factors are critical in determining the need for adjuvant chemotherapy following radical cystectomy, as they significantly affect prognosis and the risk of recurrence.Inclusion criteria were: age ≥18 years, bladder cancer diagnosis confirmed by WHO classification, availability of complete clinical data, and no prior treatment with radiotherapy, chemotherapy, or immunotherapy before surgery. Exclusion criteria were: partial resection, non-urothelial carcinoma, incomplete data, or prior treatments. This study was approved by the Ethics Committees of both participating hospitals, and informed consent was obtained from all patients. To address missing data, variables with less than 20% missingness were imputed using the K-Nearest Neighbors (KNN) method, while those with more than 20% missingness were excluded.

### 2.2 Statistical analysis and model development

Categorical variables were compared using Pearson’s chi-square test. To address class imbalances, an undersampling strategy was applied. The dataset was split into training and internal validation cohorts using five-fold cross-validation.For feature selection, we applied LASSO regression to reduce dimensionality. In particular, we applied LASSO regression to select key pathological features such as pathological stage, tumor grade, surgical margin status, and evidence of vascular/perineural invasion. These features were identified as significant predictors of adjuvant therapy response.Nine machine learning algorithms were used to develop the model: XGBoost, SVM, MLP, KNN, logistic regression, LASSO regression, decision tree (DT), GBM, and random forest (RF). The choice of using nine machine learning algorithms instead of a single one was to increase model robustness and reduce overfitting. Different algorithms have varying strengths, which allows us to capture multiple complex patterns and non-linear relationships within the data. The comparison between these algorithms ensures that the final model selected has strong generalizability and is less susceptible to overfitting.By integrating multi-dimensional data, including clinical, pathological, and molecular features, machine learning techniques enable personalized treatment predictions that account for various tumor characteristics, thereby enhancing the selection process for adjuvant chemotherapy.Model performance was evaluated using AUC-ROC, sensitivity, specificity, recall, F1-score, and accuracy. Clinical applicability was assessed using decision curve analysis (DCA), calibration plots, and clinical impact curves (CICs).SHAP analysis was performed to evaluate the contribution of each feature to the model, with summary and force plots generated for interpretability. Statistical analyses were conducted using Python, with a two-tailed p-value < 0.05 considered statistically significant.

For a more detailed description of the technical processes and calculations used, including the LASSO regression for feature selection, the machine learning algorithms employed, model performance evaluation metrics, and the SHAP analysis for model interpretability, please refer to **Supplementary Material 1**. This **Supplementary Material** includes comprehensive explanations of the methodologies and the Python code used for data preprocessing, model training, and evaluation.

## 3 Result

### 3.1 Identification of prognostic factors and construction of a predictive nomogram model for bladder cancer

Univariate analysis of the enrolled clinical features revealed significant differences in multiple clinical and molecular parameters between responders (label = 1) and non-responders (label = 0) to postoperative adjuvant therapy. Molecular markers such as Uroplakin III (p < 0.001), GATA3 (p < 0.001), CK20 (p = 0.010), and CK7 (p < 0.001) were significantly overexpressed in the responder group. In addition, immune and oncogenic markers including P63, P53, AR, and PD-L1 were also markedly upregulated among responders (all p < 0.01), suggesting their potential involvement in modulating treatment response. Several pathological features, including perineural invasion, vascular invasion, M stage, and surgical margin status, were more frequently observed in responders (all p < 0.01). Furthermore, squamous and sarcomatoid differentiation, tumor grade, and histological subtypes were significantly enriched in the responder cohort (all p < 0.001), indicating that tumors with more aggressive or immunogenic characteristics may be more sensitive to adjuvant therapy.Notably, HER2 overexpression (score 2 or 3) was more prevalent in responders than in non-responders (25% vs. 12%, p < 0.001), highlighting its potential role as a predictive biomarker for treatment response. Similarly, advanced nodal stage (N stage ≥ 1) was more common in responders (38% vs. 12%, p < 0.001), suggesting that patients with more advanced disease may derive greater benefit from adjuvant interventions. Tumor grade was also significantly higher among responders, with 96% classified as grade 1 or 2 (p < 0.001), further supporting the notion that high-grade tumors may be more responsive to therapy (**Table 1**).

**Table 1:** Baseline clinical, pathological, and molecular characteristics between responders and non-responders to postoperative adjuvant therapy in bladder.

Characteristic	0 N = 611^1^	1 N = 421^1^	p-value^2^
Gender			0.6
1	523 (86%)	356 (85%)	
2	88 (14%)	65 (15%)	
Age	305 (50%)	234 (56%)	0.073
Ethnicity	75 (12%)	65 (15%)	0.14
Smoking	287 (47%)	219 (52%)	0.11
Alcohol Consumption	182 (30%)	132 (31%)	0.6
Diabetes	68 (11%)	44 (10%)	0.7
Frequent Urination	191 (31%)	150 (36%)	0.14
Urinary Urgency	167 (27%)	119 (28%)	0.7
Dysuria	81 (13%)	80 (19%)	0.012
Pain	107 (18%)	93 (22%)	0.067
Urinary Hesitancy	81 (13%)	67 (16%)	0.2
Urinary Cytology	48 (7.9%)	47 (11%)	0.071
History of Prior Surgery	102 (17%)	91 (22%)	0.046
Urop0III	495 (81%)	278 (66%)	<0.001
GATA3	560 (92%)	347 (82%)	<0.001
CK20	370 (61%)	221 (52%)	0.010
CK7	569 (93%)	363 (86%)	<0.001
CK5/6	389 (64%)	233 (55%)	0.007
P63	522 (85%)	318 (76%)	<0.001
P53	359 (59%)	279 (66%)	0.015
AR	454 (74%)	278 (66%)	0.004
Perineural Invasion	104 (17%)	188 (45%)	<0.001
Vascular Invasion	155 (25%)	257 (61%)	<0.001
M stage	11 (1.8%)	63 (15%)	<0.001
CIS	45 (7.4%)	45 (11%)	0.063
Surgical Margins	42 (6.9%)	52 (12%)	0.003
Squamous Differentiation	81 (13%)	102 (24%)	<0.001
Glandular Differentiation	19 (3.1%)	23 (5.5%)	0.060
Neuroendocrine Differentiation	3 (0.5%)	7 (1.7%)	0.10
Sarcomatoid Differentiation	2 (0.3%)	8 (1.9%)	0.019
Histological Type			<0.001
0	590 (97%)	378 (90%)	
1	10 (1.6%)	18 (4.3%)	
2	11 (1.8%)	25 (5.9%)	
Duration of Smoking			0.3
0	324 (53%)	202 (48%)	
1	78 (13%)	53 (13%)	
2	76 (12%)	65 (15%)	
3	133 (22%)	101 (24%)	
Duration of dringking			>0.9
0	429 (70%)	289 (69%)	
1	53 (8.7%)	36 (8.6%)	
2	35 (5.7%)	25 (5.9%)	
3	94 (15%)	71 (17%)	
Blood Pressure			0.7
0	465 (76%)	318 (76%)	
1	114 (19%)	85 (20%)	
2	19 (3.1%)	13 (3.1%)	
3	13 (2.1%)	5 (1.2%)	
Hematuria			0.063
0	89 (15%)	77 (18%)	
1	32 (5.2%)	12 (2.9%)	
2	490 (80%)	332 (79%)	
PD1L1			<0.001
0	252 (41%)	227 (54%)	
1	286 (47%)	156 (37%)	
2	73 (12%)	38 (9.0%)	
HER-2			<0.001
0	288 (47%)	278 (66%)	
1	85 (14%)	39 (9.3%)	
2	132 (22%)	42 (10.0%)	
3	106 (17%)	62 (15%)	
N			<0.001
0	535 (88%)	263 (62%)	
1	49 (8.0%)	75 (18%)	
2	17 (2.8%)	46 (11%)	
3	10 (1.6%)	37 (8.8%)	
Grade			<0.001
0	147 (24%)	17 (4.0%)	
1	444 (73%)	358 (85%)	
2	20 (3.3%)	46 (11%)	

**Figure 2:**
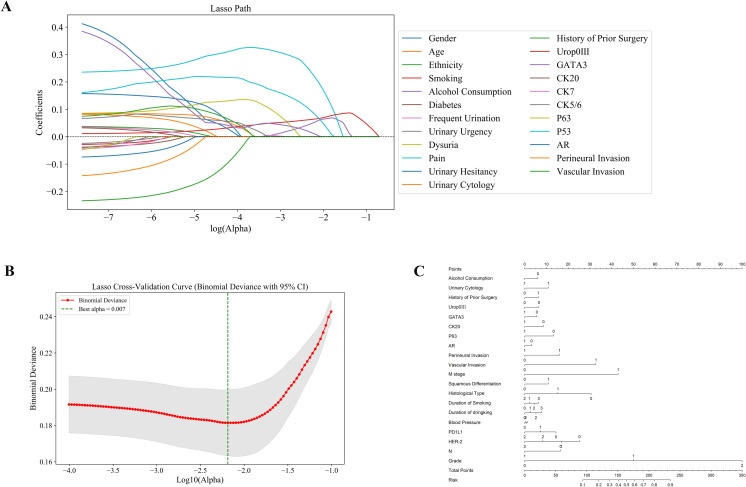
Identification of predictive features using LASSO regression and construction of the nomogram model. **(A)** LASSO coefficient profiles: Displays how the coefficients of 28 features shrink with increasing penalty, identifying key predictors associated with treatment response. **(B)** Cross-validation plot: The optimal lambda (λ = 0.007) was selected using 10-fold cross-validation to minimize binomial deviance. **(C)** Nomogram model: A predictive nomogram was developed based on selected clinical, pathological, and molecular features to estimate individual response probabilities.

Subsequently, we performed Least Absolute Shrinkage and Selection Operator (LASSO) regression analysis on the clinical variables of patients who underwent radical cystectomy, enabling the systematic identification of key predictors associated with treatment response. This approach effectively reduced model complexity and minimized potential overfitting (**Figures 2A, B**). Based on the selected variables, we developed a predictive nomogram incorporating demographic features, tumor morphology, and molecular biomarkers to generate individualized predictions of treatment response (**Figure 2C**). The selected predictors included clinical and demographic variables such as alcohol consumption, urine cytology, history of prior surgery, duration of smoking and drinking, and blood pressure, as well as histopathological features (e.g., vascular and perineural invasion, tumor stage and grade) and molecular markers (e.g., Uroplakin III, GATA3, CK20, AR, PD-L1, and HER2 expression). Each variable was weighted according to its contribution to the model, allowing for intuitive quantification of individual risk scores.The constructed nomogram exhibited excellent predictive performance, effectively integrating tumor morphology, patient demographics, and molecular biomarker expression. Collectively, these findings underscore the potential of our integrated predictive model in accurately assessing postoperative adjuvant therapy response in bladder cancer, providing a valuable tool for personalized clinical decision-making (**Figure 2**).

### 3.2 Model construction and validation for predicting adjuvant therapy response in bladder cancer patients

**Figure 3:**
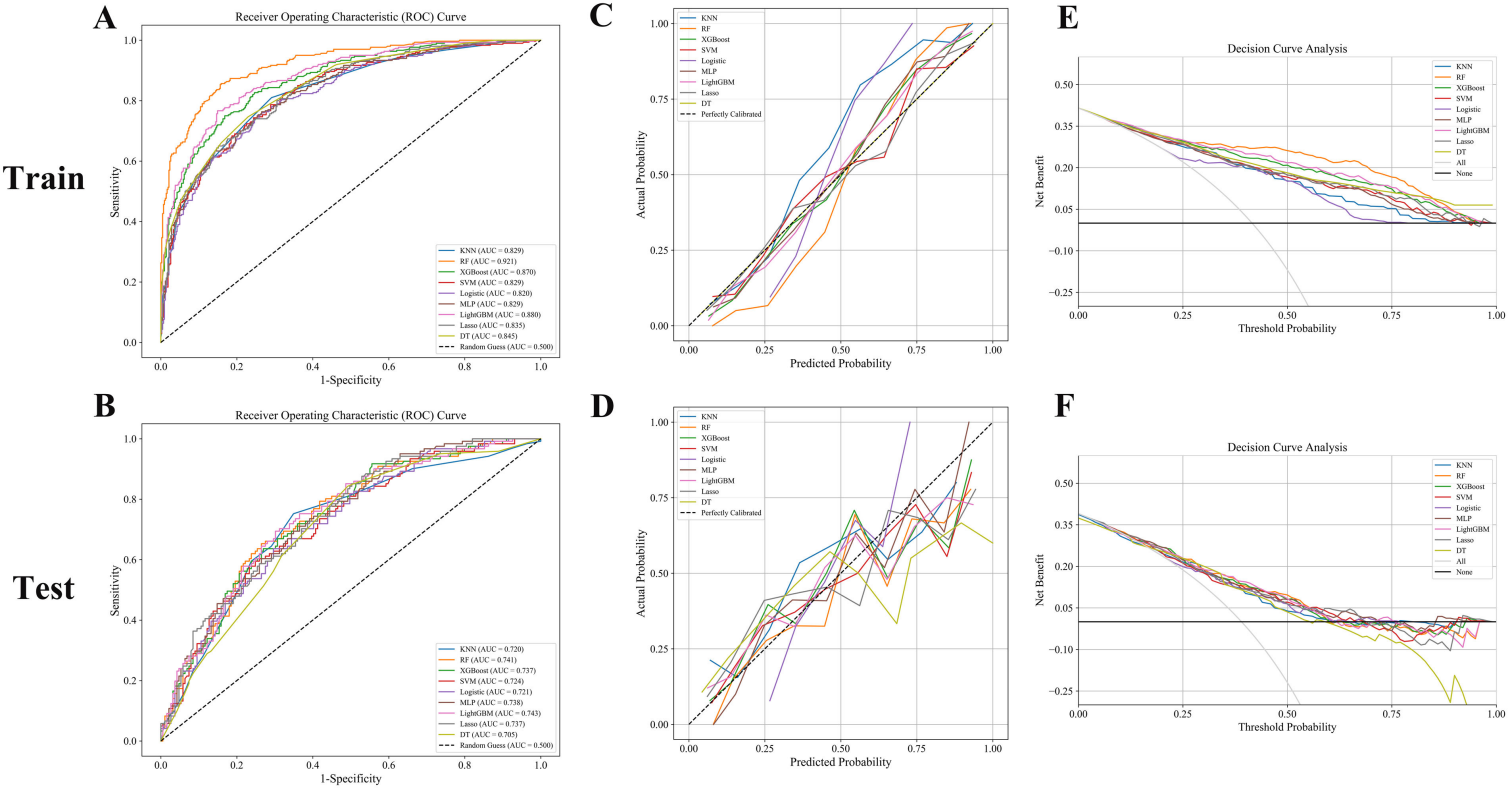
**(A, B)** ROC curves: RF, LightGBM, and XGBoost models achieved superior AUCs, indicating excellent classification performance. **(C, D)** Calibration curves: Good agreement was observed in the training set, while the test set showed greater variability. **(E, F)** Decision curve analysis: RF and LightGBM consistently provided the highest net benefit across decision thresholds.

In this study, eight machine learning algorithms were employed to evaluate the predictive performance of postoperative adjuvant therapy response in bladder cancer patients. The models included K-nearest neighbors (KNN), random forest (RF), extreme gradient boosting (XGBoost), support vector machine (SVM), logistic regression, multilayer perceptron (MLP), Light Gradient Boosting Machine (LightGBM), LASSO regression, and decision tree (DT), with assessments conducted on both training and testing cohorts (**Figure 3**). In the training set, the RF model demonstrated the highest overall performance, with an area under the curve (AUC) of 0.921, accuracy of 0.846, and F1-score of 0.847, indicating excellent discriminatory power and a balanced trade-off between precision and recall. Both LightGBM and XGBoost also achieved favorable results, with AUC values of 0.880 and 0.870, respectively. The KNN model yielded the highest specificity (0.953), while RF achieved the highest negative predictive value (NPV = 0.875) and Youden index (0.706), suggesting an optimal balance between sensitivity and specificity. The RF model also showed the highest Kappa coefficient (0.685), underscoring its stability and agreement with actual outcomes (**Figure 3A**, **Tables 2**, **3**).

**Table 2:** Comparison of predictive performance across nine machine learning models in the training and testing sets for bladder cancer adjuvant therapy response.

Comparison of results across models									
	KNN	RF	XGB	SVM	LR	MLP	Lightgbm	Lasso	DT
Accuracy	0.737	0.846	0.794	0.751	0.735	0.758	0.802	0.759	0.766
PPV	0.771	0.847	0.793	0.749	0.751	0.758	0.801	0.757	0.764
Recall	0.737	0.846	0.794	0.751	0.735	0.758	0.802	0.759	0.766
F1_score	0.713	0.847	0.791	0.747	0.719	0.752	0.8	0.757	0.763
AUC	0.829	0.921	0.87	0.829	0.82	0.829	0.88	0.835	0.845
Specificity	0.953	0.86	0.872	0.841	0.917	0.863	0.867	0.827	0.841
NPV	0.703	0.875	0.795	0.759	0.713	0.757	0.808	0.776	0.777
Youden Index	0.689	0.706	0.666	0.592	0.653	0.62	0.669	0.586	0.607
Kappa	0.416	0.685	0.567	0.475	0.422	0.486	0.586	0.497	0.51
Accuracy	0.648	0.706	0.69	0.681	0.674	0.69	0.694	0.674	0.645
PPV	0.645	0.706	0.683	0.673	0.672	0.683	0.688	0.666	0.641
Recall	0.648	0.706	0.69	0.681	0.674	0.69	0.694	0.674	0.645
F1_score	0.596	0.706	0.683	0.672	0.642	0.679	0.689	0.666	0.643
AUC	0.72	0.741	0.737	0.724	0.721	0.738	0.743	0.737	0.705
Specificity	0.915	0.762	0.81	0.804	0.894	0.831	0.788	0.794	0.73
NPV	0.65	0.758	0.718	0.71	0.676	0.71	0.73	0.708	0.701
Youden Index	0.564	0.468	0.5	0.485	0.568	0.521	0.482	0.468	0.375
Kappa	0.166	0.382	0.325	0.303	0.247	0.317	0.341	0.291	0.245

**Table 3:** Comparison of confusion matrix outputs for nine machine learning models in the training and testing sets.

Comparison of confusion matrix results across models									
Train					Test				
	TN	FP	FN	TP		TN	FP	FN	TP
KNN	402	20	170	130	KNN	173	16	93	28
RF	363	59	52	248	RF	144	45	46	75
XGB	368	54	95	205	XGB	153	36	60	61
SVM	355	67	113	187	SVM	152	37	62	59
LR	387	35	156	144	LR	169	20	81	40
MLP	364	58	117	183	MLP	157	32	64	57
LightGBM	366	56	87	213	LightGBM	149	40	55	66
Lasso	349	73	101	199	Lasso	150	39	62	59
DT	355	67	102	198	DT	138	51	59	62

However, model performance declined in the testing set, indicating reduced generalizability and a potential risk of overfitting (**Figures 3C, D**). RF retained the best AUC (0.741), followed closely by LightGBM (0.743) and XGBoost (0.737). Accuracy in the testing cohort ranged from 0.645 (DT) to 0.706 (RF), with moderate F1-scores across all models, further suggesting some degree of overfitting (**Figure 3B**, **Tables 2**, **3**). Notably, specificity remained high in logistic regression and KNN (≥0.89), despite the overall performance decline.

Decision curve analysis (DCA) revealed that XGBoost and LightGBM models provided substantial net clinical benefit across a wide range of threshold probabilities in the training set. In the testing set, most models still demonstrated moderate clinical utility at low to intermediate risk thresholds, though the net benefit was diminished (**Figures 3E, F**). Taken together, these results suggest that the developed machine learning models—particularly the RF model—hold considerable potential for predicting responses to adjuvant therapy in bladder cancer. Nonetheless, further external validation is necessary to enhance model robustness and ensure its reliability in real-world clinical settings.

### 3.3 Clinical applicability of machine learning models evaluated by clinical impact curves

**Figure 4:**
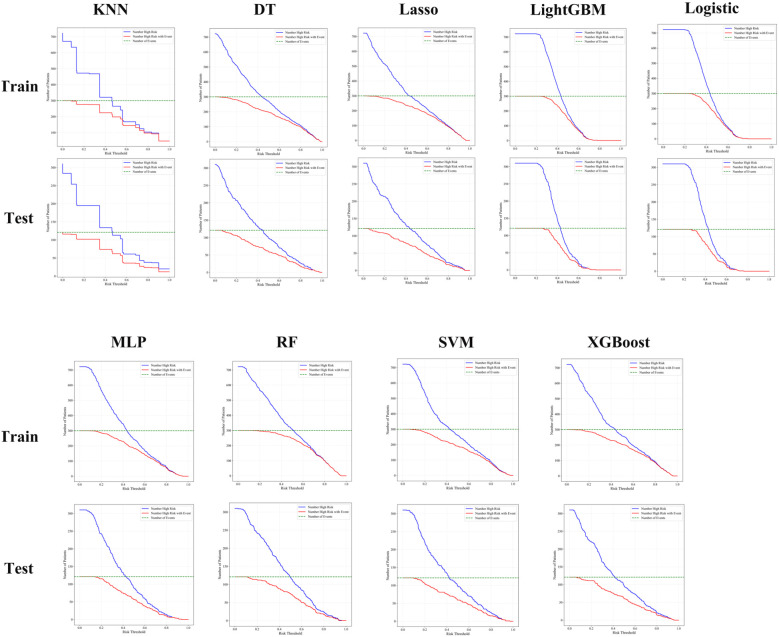
Clinical impact curve analysis showed that random forest, RF, LightGBM, and XGBoost models consistently identified high-risk patients with better accuracy and clinical utility across both training and testing cohorts.

Clinical impact curve (CIC) analysis was performed to evaluate the clinical applicability of eight machine learning models—K-nearest neighbors (KNN), random forest (RF), extreme gradient boosting (XGBoost), support vector machine (SVM), logistic regression, multilayer perceptron (MLP), LightGBM, LASSO regression, and decision tree (DT)—in both the training and testing cohorts (**Figure 4**). The CICs demonstrated considerable variability in the ability of these models to accurately identify high-risk patients across different risk thresholds. Notably, the RF, XGBoost, and LightGBM models exhibited superior and more stable predictive performance, more accurately reflecting the number of true positive cases and effectively distinguishing between high- and low-risk patient groups. These findings highlight the promising clinical utility of RF, XGBoost, and LightGBM in predicting responses to postoperative adjuvant therapy in bladder cancer patients. In future clinical applications, these robust models should be prioritized, and careful selection of the optimal risk threshold will be essential to achieving maximum clinical benefit (**Figure 4**).

### 3.4 Feature importance interpretation using SHAP analysis

**Figure 5:**
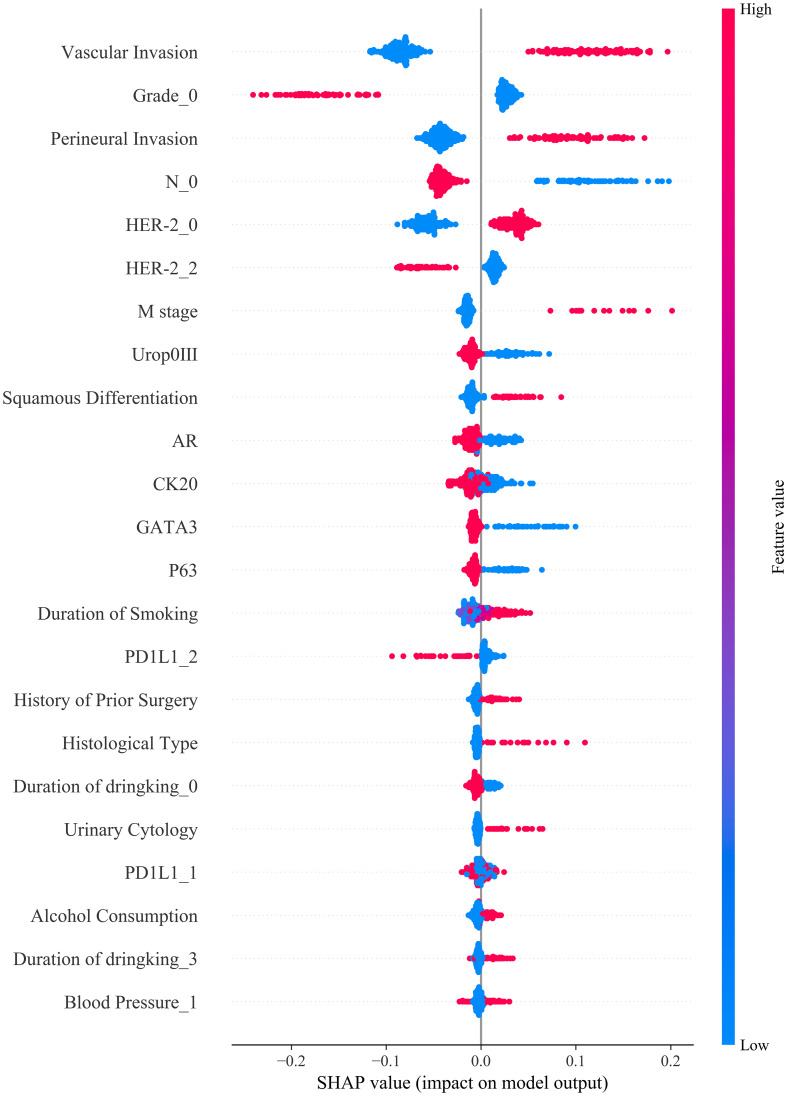
SHAP summary plot illustrating the contribution of each feature to model output: Vascular invasion, tumor grade, perineural invasion, and nodal stage were the most influential predictors of treatment response, with higher feature values (in red) generally associated with increased predicted risk.

To interpret the relative contributions of each predictive feature within our machine learning model, we conducted Shapley Additive Explanations (SHAP) analysis (**Figure 5**). The SHAP swarm plot clearly illustrates how individual clinical, demographic, and molecular variables influenced model predictions. Vascular invasion, tumor grade, perineural invasion, and lymph node involvement (N stage) were among the most influential clinical and pathological predictors, positively associated with higher risk predictions. Additionally, molecular markers including HER-2, PD-L1, CK20, GATA3, and P63 showed significant impacts, with elevated expressions correlating strongly with adverse outcomes. Demographic and behavioral features, such as alcohol consumption, smoking duration, and blood pressure, also contributed meaningfully to the prediction outcomes, highlighting the multifactorial nature of bladder cancer prognosis. Collectively, SHAP analysis provided comprehensive insights into how individual clinical, pathological, and molecular features influenced model predictions, thus enhancing model interpretability and clinical transparency (**Figure 5**). These findings support the clinical relevance of the selected variables and emphasize their utility for personalized prognosis and therapeutic decision-making in bladder cancer.

## 4 Discussion

In recent years, artificial intelligence (AI) has attracted increasing attention in the field of personalized medicine for bladder cancer, particularly in predicting responses to postoperative adjuvant therapy. Compared with traditional statistical methods, machine learning (ML) techniques have demonstrated greater potential in handling complex, high-dimensional data, enabling more precise analysis for individualized treatment planning (16). Advances in AI have facilitated its application in bladder cancer diagnosis, staging, and therapeutic response prediction, thus supporting clinical decision-making.

Beyond therapeutic prediction, AI has also been applied to detect genetic alterations such as FGFR3 mutations directly from histopathological images. AI systems have shown promising results in identifying FGFR3 mutation status from routine histological slides, offering a valuable pre-screening tool for subsequent molecular testing (17). Unlike traditional models such as Cox regression and Kaplan-Meier survival analysis—which rely on univariate or linear relationships—ML algorithms such as XGBoost and LightGBM are better suited to capturing non-linear interactions among high-dimensional variables, thereby improving predictive performance (18–20).

In this study, we integrated tumor morphological features, patient demographics, and molecular marker expression to construct a comprehensive predictive model for postoperative adjuvant therapy response in bladder cancer. Using LASSO regression, clinically meaningful variables were systematically selected to reduce model complexity and minimize the risk of overfitting. These features included tumor aggressiveness markers (e.g., vascular and perineural invasion, histological subtype, tumor grade), molecular biomarkers (e.g., HER2, PD-L1, CK20, GATA3, Uroplakin III), and behavioral factors (e.g., smoking and alcohol consumption). The incorporation of these multidimensional variables enhanced the model’s ability to more accurately and comprehensively predict treatment responses, aligning with the needs of real-world clinical settings.

During model development and validation, we systematically compared nine common ML algorithms, including XGBoost, LightGBM, SVM, and logistic regression. ML methods have increasingly demonstrated value in clinical prediction tasks across various diseases. For example, SVM and boosting algorithms have shown excellent performance in cardiovascular disease prediction (21), while ensemble models such as RF and XGBoost achieved AUCs of 0.96 and 0.97, respectively, in pneumonia diagnosis (22). RF also outperformed other algorithms in predicting postoperative delirium (AUC = 0.994) (23), and in breast cancer survival prediction, where a tuned RF (TRF) model reached 96% accuracy and sensitivity (24). In bladder cancer, deep learning has been used to recalibrate the CUETO and EORTC tools for recurrence and progression risk, demonstrating better performance than conventional methods (25). Recent studies have further investigated the role of machine learning in bladder cancer patient stratification, highlighting the potential of ML algorithms to enhance the accuracy and precision of patient risk assessments (26). These findings are consistent with our results and reinforce the clinical applicability of ML models in the personalized treatment of bladder cancer.

Encouragingly, in our study, the random forest (RF) model achieved the highest predictive performance in the training cohort (AUC = 0.921) and maintained good generalizability in the external validation cohort (AUC = 0.741). Although LightGBM and XGBoost also performed well during training, their performance declined in external validation, suggesting potential susceptibility to data heterogeneity. RF’s robustness in medical prediction tasks has been consistently demonstrated across studies due to its ability to combine multiple decision trees and capture complex non-linear interactions. Decision curve analysis (DCA) further confirmed the strong clinical net benefit of XGBoost and LightGBM across a wide range of threshold probabilities, supporting their practical utility in clinical settings.

In this study, several important predictors were identified using SHAP analysis, but the clinical biological rationale behind these features has not been fully explained. PD-L1 high expression plays a crucial role in tumor immune evasion by binding to the PD-1 receptor on T cells, allowing the tumor to escape immune surveillance. High PD-L1 expression has also been associated with better responses to immune checkpoint inhibitors like Pembrolizumab and Atezolizumab. Thus, PD-L1 expression directly impacts the prediction of postoperative adjuvant therapy efficacy. HER2 overexpression promotes tumor cell proliferation and survival by activating key signaling pathways such as PI3K/Akt and MAPK, making it a critical factor for bladder cancer aggressiveness and prognosis. In our study, HER2 overexpression was identified as an important predictor of poor prognosis. Vascular invasion and perineural invasion are pathological features that suggest tumor aggression. Vascular invasion, indicating the potential for distant metastasis, and perineural invasion, associated with worsened prognosis and pain, are crucial in predicting treatment responses and patient outcomes.

To better integrate our model into real-world clinical workflows, we recommend embedding it into Clinical Decision Support Systems (CDSS) through Electronic Medical Records (EMR) for real-time risk assessment and personalized treatment recommendations. This integration would assist clinicians in quickly evaluating postoperative adjuvant therapy responses in bladder cancer patients, while optimizing treatment plans and resource allocation. Future studies should focus on multi-center validation, long-term follow-up, and regular model updates to ensure its sustained accuracy and clinical applicability.

To improve the generalizability of our model and address the reduced performance in the external validation cohort, several factors should be considered in future work. Data heterogeneity between the external cohort and training set may affect predictive accuracy. Differences in sample size and feature distribution could also lead to less robust predictions. Overfitting, a common issue with models performing well in training but poorly in new data, may have contributed to these discrepancies. Future studies should incorporate multi-center validation, longitudinal data, and model regularization techniques (e.g., Elastic Net) to enhance robustness, reduce overfitting, and improve generalizability. These steps will optimize the model’s clinical applicability.

To further interpret model behavior, SHAP (SHapley Additive exPlanations) analysis was employed to visualize the contribution of each feature to model predictions. Tumor features such as grade and vascular invasion were identified as core predictors of poor prognosis, and high expression of HER2 and PD-L1 was strongly associated with adverse outcomes. SHAP also provided individualized explanations for prediction results, enhancing model transparency and clinical trust. The clinical significance of HER2 and PD-L1 is increasingly recognized in postoperative, chemotherapy, and immunotherapy contexts. PD-L1, in particular, is a widely studied predictive biomarker for immune checkpoint inhibitors (ICIs) across cancers, including bladder cancer, where its high expression is associated with advanced pathological stage and better response to therapies like pembrolizumab and atezolizumab (27, 28). Nevertheless, its predictive value remains controversial, as some patients with low PD-L1 expression still benefit from ICIs, while not all high expressers respond (29). HER2, though less explored in bladder cancer than in breast cancer, represents another promising therapeutic target. Incorporating HER2 and PD-L1 into prognostic models may enhance treatment stratification and aligns with the paradigm of precision oncology (30).

Our study also considered lifestyle factors such as smoking and drinking history, which have been associated with patient prognosis and are consistent with prior studies on the interaction between tumor biology and environmental/genetic factors (31, 32). Clinical impact curves (CICs) provided additional validation, showing that RF, XGBoost, and LightGBM models could more accurately identify high-risk individuals for actual adverse events and support effective threshold selection in clinical decision-making. In contrast, models such as DT and LASSO underperformed, indicating limited adaptability in diverse clinical scenarios.

From a clinical application perspective, our findings can support personalized treatment strategies. For high-risk patients identified by the model, clinicians could consider more aggressive adjuvant regimens (e.g., combined chemotherapy and immunotherapy), whereas low-risk patients may benefit from de-escalated treatment, thus reducing unnecessary toxicity. DCA results showed that XGBoost and LightGBM models offer higher net benefit at low to moderate risk thresholds, suggesting their utility in early clinical decision-making. In the future, these models could be incorporated into a clinical decision support system (CDSS) embedded within electronic medical records (EMR) to enable automated, real-time risk assessment and streamline clinical workflows.

In conclusion, the integrated predictive model developed in this study effectively enhances the accuracy and clinical applicability of predicting postoperative adjuvant therapy response in bladder cancer by combining clinicopathological and molecular biomarker information. Although the model exhibited slightly reduced performance in external validation, indicating the need for improved generalizability, it holds promising translational value. Future studies should involve larger, multi-center datasets for external validation and aim to optimize model robustness, ultimately contributing to more precise and personalized treatment strategies for bladder cancer patients.

## 5 Conclusion

In this study, we successfully developed a machine learning model that integrates tumor morphological features, demographic variables, and molecular marker expression to predict the response of bladder cancer patients to postoperative adjuvant therapy. The model demonstrated excellent performance in the training cohort; however, a decline in performance was observed in the testing cohort, indicating that further validation is needed to improve its generalizability. SHAP analysis identified key predictive features, including vascular invasion, perineural invasion, and the expression of HER2 and PD-L1. Decision curve analysis (DCA) revealed a favorable clinical net benefit within the moderate risk threshold range. Future research should focus on external validation using multi-center datasets and explore the development of dynamic prediction models based on longitudinal data to enhance robustness and clinical utility.

## Data Availability

The original contributions presented in the study are included in the article/**Supplementary Material**. Further inquiries can be directed to the corresponding authors.
