# Supplementary material for: Explainable machine learning model predicts response to adjuvant therapy after radical cystectomy in bladder cancer
Source: Front Oncol. 2025 Oct 31;15:1664965. doi: 10.3389/fonc.2025.1664965 (PMC12615217; doi:10.3389/fonc.2025.1664965)
Supplement: Supplementary file 1 [file Supplementaryfile1.docx]

**Supplementary Material 1**

**1. LASSO Regression and Feature Selection**

In this study, we employed Least Absolute Shrinkage and Selection Operator (LASSO) regression to select key features that are predictive of postoperative adjuvant therapy response in bladder cancer. LASSO regression utilizes L1 regularization, which encourages sparsity in the coefficients of the model, effectively shrinking some coefficients to zero and thus removing less important features. This approach helped to reduce the dimensionality of the feature set while preserving the most clinically relevant variables. The process involved performing 10-fold cross-validation to determine the optimal regularization parameter (λ), which was selected to minimize binomial deviance and prevent overfitting. The features selected by LASSO were further utilized in the machine learning models for training and validation. These included tumor morphology, molecular biomarkers, and clinical variables such as perineural invasion, vascular invasion, and expression of immune-related markers like PD-L1 and HER2. The coefficient profiles for LASSO regression were visually inspected to identify the most influential predictors.

**2. Machine Learning Algorithms and Hyperparameter Optimization**

To develop the predictive model, we tested nine machine learning (ML) algorithms: extreme gradient boosting (XGBoost), support vector machine (SVM), multilayer perceptron (MLP), k-nearest neighbors (KNN), logistic regression, LASSO regression, decision tree (DT), gradient boosting machine (GBM), and random forest (RF). The decision to use nine algorithms was made to ensure robustness and accuracy by exploring different model architectures that handle high-dimensional data in diverse ways. By using multiple algorithms, we were able to capture various patterns and non-linear relationships, which could be missed by a single model. Furthermore, this multi-algorithm approach reduces the risk of overfitting and ensures better generalizability of the final model. For each algorithm, we optimized hyperparameters using a grid search strategy, where a predefined set of hyperparameters was tested, and the combination that resulted in the best performance was selected. Key hyperparameters tuned include learning rates, maximum depth of trees, regularization parameters, and number of estimators. Grid search was conducted on the training set using 5-fold cross-validation. Model performance was evaluated using multiple metrics, including area under the receiver operating characteristic curve (AUC-ROC), accuracy, sensitivity, specificity, recall, and F1-score. This process ensured that the selected model achieved a balanced trade-off between sensitivity and specificity, with minimal overfitting.

**3. Model Performance Metrics and Evaluation**

The performance of the machine learning models was assessed using several metrics to evaluate both discrimination and calibration. AUC-ROC was used to evaluate the model’s discriminatory ability, while accuracy, sensitivity, specificity, recall, and F1-score were calculated to provide insight into the model’s overall performance in distinguishing between responders and non-responders to postoperative adjuvant therapy. Decision curve analysis (DCA) was performed to assess the clinical net benefit of the model across different threshold probabilities, providing a measure of the model’s clinical utility. Calibration plots were generated to evaluate how well the predicted probabilities of response matched the actual observed outcomes. Additionally, clinical impact curves (CICs) were plotted to identify the optimal decision thresholds, ensuring that the model’s predictions were not only statistically significant but also useful for guiding clinical decision-making. The calibration and DCA plots were constructed using the R packages ggplot2 and dca, with thresholds set at various levels to identify the best trade-offs between sensitivity and specificity.

**4. SHAP Analysis for Model Interpretability**

To enhance model interpretability, we utilized SHapley Additive exPlanations (SHAP), a method that breaks down a prediction to show the contribution of each feature to the final outcome. SHAP summary plots were generated to visualize the overall contribution of each feature to the model’s predictions. These plots allow us to identify which features, such as vascular invasion, tumor grade, and PD-L1 expression, had the most significant impact on the model’s predictions. Additionally, SHAP force plots were generated to provide individualized explanations for specific predictions, allowing clinicians to understand why a particular patient was classified as a responder or non-responder to adjuvant therapy. This transparency helps to build trust in the model’s predictions and allows for easier integration into clinical practice. We found that HER2, vascular invasion, and PD-L1 were the most influential predictors, with higher values of these features leading to predictions of poor prognosis.

**5. Python Code and Data Preprocessing Details**

The data preprocessing and machine learning models were implemented in Python (version 3.8) using key libraries such as scikit-learn, XGBoost, and LightGBM. The preprocessing steps involved the following:

**1.Data Imputation:** Missing data were handled using the K-Nearest Neighbors (KNN) method for variables with less than 20% missingness. For features with greater than 20% missingness, these were excluded from the analysis to prevent bias.

**2.Feature Scaling:** All continuous variables were scaled using Min-Max scaling, ensuring that features with different units did not dominate the model.

**3.Model Implementation:** The algorithms were implemented using scikit-learn, XGBoost, and LightGBM libraries. Key hyperparameters for each model were optimized using GridSearchCV. For XGBoost and LightGBM, the number of boosting rounds, learning rate, and tree depth were optimized. For SVM and logistic regression, we fine-tuned the C parameter (regularization strength).

**4.Cross-Validation:** 5-fold cross-validation was used for training, and performance metrics were evaluated for each fold. The final model was trained on the full training dataset after hyperparameter optimization.
